# Genetic and clinical characterization of a novel *FH* founder mutation in families with hereditary leiomyomatosis and renal cell cancer syndrome

**DOI:** 10.1186/s13023-024-03017-z

**Published:** 2024-01-26

**Authors:** Ana Beatriz Sánchez-Heras, Estela Dámaso, Adela Castillejo, Mercedes Robledo, Alexandre Teulé, Conxi Lázaro, Rosario Sánchez-Martínez, Ángel Zúñiga, Adrià López-Fernández, Judith Balmaña, Luis Robles, Teresa Ramon y Cajal, M. Isabel Castillejo, Raquel Perea Ibañez, Carmen Martínez Sevila, Andrea Sánchez-Mira, Inés Escandell, Luís Gómez, Pere Berbel, José Luis Soto

**Affiliations:** 1 Cancer Genetic Counselling Unit, Medical Oncology Department, Hospital General Universitario de Elche, 03203 Elche, Alicante Spain; 2 Molecular Genetics Laboratory, Hospital General Universitario de Elche, Elche, Alicante Spain; 3 Foundation for the Promotion of Health and Biomedical Research of Valencia Region (FISABIO), FISABIO-Elche Health Department, Elche, Spain; 4 Hereditary Endocrine Cancer, Human Cancer Genetics Programme Spanish National Cancer Centre (CNIO), Madrid, Spain; 5 Centro de Investigación Biomédica en Red de Enfermedades Raras, CIBERER, 28029 Madrid, Spain; 6 Hereditary Cancer Program, Program in Molecular Mechanisms and Experimental Therapy in Oncology (Oncobell), IDIBELL, Catalan Institute of Oncology, L’Hospitalet del Llobregat, Barcelona, Spain; 7 Multidisciplinary Rare Disease Unit, Internal Medicine Department, Alicante University General Hospital, Alicante Institute for Health and Biomedical Research (ISABIAL), Alicante, Spain; 8 Clinical Genetics Unit, Hospital Politécnico y Universitario La Fe, Valencia, Spain; 9 Hereditary Cancer Genetics Group, VHIO, and Medical Oncology Department, Hospital Vall D’Hebron, Barcelona, Spain; 10 Medical Oncology Department. Hospital 12 de Octubre, Madrid, Spain; 11 Familiar Cancer Clinic, Medical Oncology Department, Santa Creu i Sant Pau Hospital, Barcelona, Spain; 12 Servicio de Dermatología, Hospital General Universitario de Elda, Elda, Alicante Spain; 13 Urology Department, Hospital General Universitario de Elche, Elche, Alicante Spain; 14 Departamento de Histología y Anatomía, Facultad de Medicina, Universidad Miguel Hernández, Sant Joan d’Alacant, Alicante Spain

**Keywords:** Haplotypes, Founder mutation, Common ancestor, Hereditary leiomyomatosis, *FH*, Renal cell cancer, Fumarate hydratase, Leiomyoma

## Abstract

**Background:**

Hereditary leiomyomatosis and renal cell cancer syndrome is a rare autosomal dominant hereditary syndrome. Previously, we published the largest cohort of *FH* mutation carriers in Spain and observed a highly recurrent missense heterozygous variant, *FH*(NM_000143.4):c.1118A > G p.(Asn373Ser), in 104 individuals from 31 apparently unrelated families. Here, we aimed to establish its founder effect and characterize the associated clinical phenotype.

**Results:**

Haplotype analysis confirmed that families shared a common haplotype (32/38 markers) spanning 0.61–0.82 Mb, indicating this recurrent variant was inherited from a founder ancestor. Cutaneous and uterine leiomyomatosis were diagnosed in 64.6% (64/99) and 98% (50/51) of patients, respectively, and renal cell cancer was present in 10.4% (10/96). The pathogenic *FH*_c.1118A > G variant is a Spanish founder mutation that originated 12–26 generations ago. We estimate that the variant may have appeared between 1370 and 1720. Individuals carrying this founder mutation had similar frequency of renal cell cancer and a higher frequency of renal cysts and leiomyomas than those in other cohorts of this syndrome.

**Conclusions:**

In the Spanish province of Alicante there is a high prevalence of HLRCC because of the founder mutation *FH* c.1118A > G; p.(Asn373Ser). The characterization of founder mutations provides accurate and specific information regarding their penetrance and expressivity. In individuals with suspected HLRCC from the province of Alicante, genetic testing by direct analysis of the founder *FH* c.1118A > G; p.(Asn373Ser) mutation may be a faster and more efficient diagnostic tool compared with complete gene sequencing.

**Supplementary Information:**

The online version contains supplementary material available at 10.1186/s13023-024-03017-z.

## Introduction

Hereditary leiomyomatosis and renal cell cancer (HLRCC) syndrome (OMIM 605839) was first described by Reed et al. in 1973 [1]. Tomlinson et al. confirmed that it is caused by heterozygous pathogenic variants in the gene encoding fumarate hydratase (*FH*) [2]. As a consequence, the deficient FH enzyme leads to tricarboxylic acid cycle failure and energy metabolic alterations. Heterozygous carriers may develop cutaneous leiomyomas (CLM) and uterine leiomyomas (ULM), renal cysts (RCys), and renal cell cancer (RCC) [2–8] and other tumors, such as paragangliomas or suprarenal adenomas [9, 10]. Homozygous carriers suffer from *FH* deficiency (OMIM 606812), which is a metabolic disorder presenting with severe encephalopathy and a short life expectancy [11]. Therefore, it is essential to offer genetic counseling to heterozygous carriers of mutations in *FH*.

HLRCC is considered as a rare disease with scarce data available regarding its prevalence [6, 12]. We previously published a series of 197 patients from Spain [13], in which we described 27 germline variants in *FH* and observed a highly recurrent missense variant, *FH*(NM_000143.4):c.1118A > G p.(Asn373Ser), in 104 individuals from 31 apparently unrelated families, the majority of whom were from the Alicante province in the southeast of Spain.

Recurrent variants may be generated by independent mutational events, suggesting mutation hotspots, or be inherited from a common ancestor, initially evidenced by a founder effect. Testing for founder variants affords a faster molecular diagnosis by avoiding the screening of all possible gene variants, thus rendering the process more cost-effective. Moreover, the characterization of founder mutations provides a unique model with accurate and specific information regarding the penetrance and expressivity of clinical manifestations, especially in rare diseases, such as HLRCC.

Our aim was to establish the founder effect of the missense pathogenic *FH* c.1118A > G variant and to describe the clinical characteristics associated with its heterozygous carriers.

## Patients and methods

### Patients and data collection

Carriers and obligate carriers of the *FH* c.1118A > G variant from families with a genetic diagnosis of HLRCC from different hospitals in Spain were included in the present study. All patients gave written informed consent for genetic testing according to the Spanish legislation. This study was approved by the Research Ethics Committee of the Hospital General Universitario de Elche on January 25, 2018 (code 42/2017).

We collected clinical information from medical records, such as the presence and age of diagnosis of CLM, ULM, RCy, and RCC. RCC was confirmed by histological examination of biopsies or resected tumors. Imaging procedures, such as CT, magnetic resonance imaging, or ultrasound, were used to diagnose RCy. To assess the clinical phenotype associated with this variant and to identify genotype–phenotype correlations, we compared our cohort with individuals carrying loss-of-function (LoF) variants, including large deletions and nonsense, frameshift, and splicing pathogenic variants previously published by our group [13].

### Samples

Twenty-seven unrelated *FH*(NM_000143.4):c.1118A > G heterozygous carriers were selected for the haplotype study. In addition, 20 noncancer healthy controls with confirmed absence of this variant were selected as the control population. Isolation of genomic DNA from the peripheral blood of all patients included in the study was performed using a QIAamp DNA Mini Kit and QIAcube (QIAGEN, Germany) according to the manufacturer’s instructions.

### Haplotype analysis

Haplotype construction was performed using 38 polymorphic markers (microsatellite markers and single-nucleotide variants) flanking *FH* and covering nearly 14 Mb. Ten microsatellite markers covering the *FH* locus were selected from the MapViewer database. Microsatellite markers were selected based on the following criteria: 1) physical position around the *FH* locus, 2) high heterozygosity (> 0.5), and 3) inclusion of dinucleotide repeats.

In addition, 28 single-nucleotide variants located around the *FH* locus and exhibiting an allele frequency in the non-Finnish European population (GnomAD) between 0.25 and 0.75 were selected. PCR amplicons including more than one variant were designed when no evidence of linkage was found. The selected markers and their genomic position are presented in Additional file 1: Figure S1.

### Time of most recent common ancestor (TMRCA) calculation

The age of the mutation was estimated using the single marker method based on the expected decay of linkage, as previously reported [14]. Marshfield genetic distances (cM) and physical distances (Mb) were obtained from the Ensembl and UCSC databases, respectively [15, 16]. Estimation of the most recent common ancestor was performed assuming an average of 25 years between generations.

### Statistics analyses

We used the R software (R Foundation for Statistical Computing), version 3.6.0, for the statistical analyses. The qualitative variables are presented as percentages, and the continuous quantitative variables are described as the mean and standard deviation (SD) or as the median and the interquartile range (IQR). Categorical variables were compared using the chi-squared and Fisher’s exact tests and multivariate logistic regression. Odds ratio (ORs) were calculated to estimate the strength of the association between variables. The confidence level used was the 95% confidence interval (95% CI). Significance was set at *P* < 0.05. The cumulative incidence of events was estimated using the cumulative hazard function.

## Results

### Haplotype characterization

For haplotype characterization, index cases were selected from each individual family. We included 20 families from the same geographic area (Alicante province) and seven families from other Spanish regions. Moreover, we included 20 unrelated healthy controls for TMRCA calculations as nonaffected chromosome carriers.

The haplotype analysis confirmed that families shared a common haplotype (32/38 markers) spanning between 0.61 and 0.82 Mb (1.40–1.89 cM). In Table 1, we included the result of the haplotype study for the 20 index cases from the province of Alicante. The remaining seven cases from other geographical areas were studied only for the 10 microsatellites due to the limited amount of DNA available. In any case, the haplotype results of these seven cases were consistent with the results of the other 20 and did not provide any extra information (Table 1). We identified a conserved haplotype that cosegregated with the mutation and was absent in healthy controls. This result strongly suggests that the *FH* c.1118A > G variant is a founder mutation, thus ruling out the recurrent mutation hotspot hypothesis.

**Table 1:**
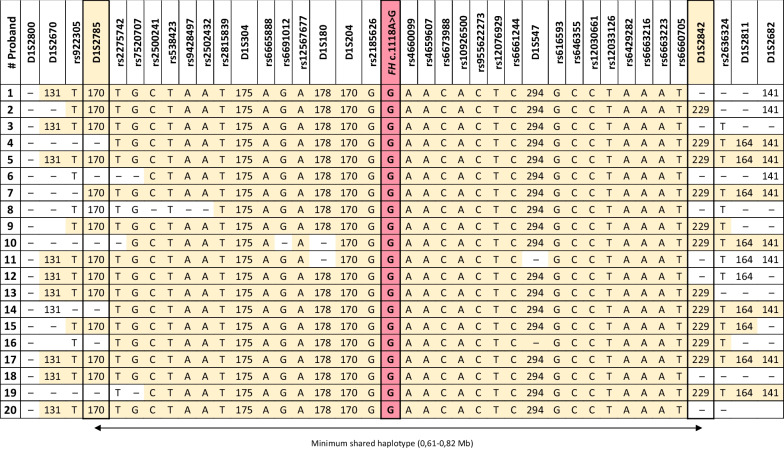
Minimum common haplotype among 20 FH c.1118A > G carrier families (the variant is highlighted in red). All carriers shared a common 0.61–0.82 Mb haplotype (yellow) delimited by the D1S2785 and D1S2842 microsatellite markers. The minimum common shared haplotype is indicated at the bottom by an arrow

The mutation age was estimated using a single marker method [17], with D1S2785 and D1S2842 as main recombinant markers, based on the genotypes observed on affected chromosomes. The estimated number of generations since the appearance of the most recent common ancestor was between 12 and 26 (Additional file 2: Table S1). Assuming 25 years per generation, the founder *FH* mutation arose between 300 and 650 years ago (ca. 1370–1720).

### Clinical findings

In total, 104 patients belonging to 31 different families were heterozygous carriers of the *FH* c.1118A > G variant; they comprised 31 index cases and 73 relatives. This group included 53 women (51%) and 51 men (49%) with a mean age of 53.3 (range, 12–91) years; 94 patients from 26 families originated from the province of Alicante in southeast Spain. The estimated prevalence of HLRCC due to founder variant in this province is 6.26/100,000 inhabitants.

**Fig. 1:**
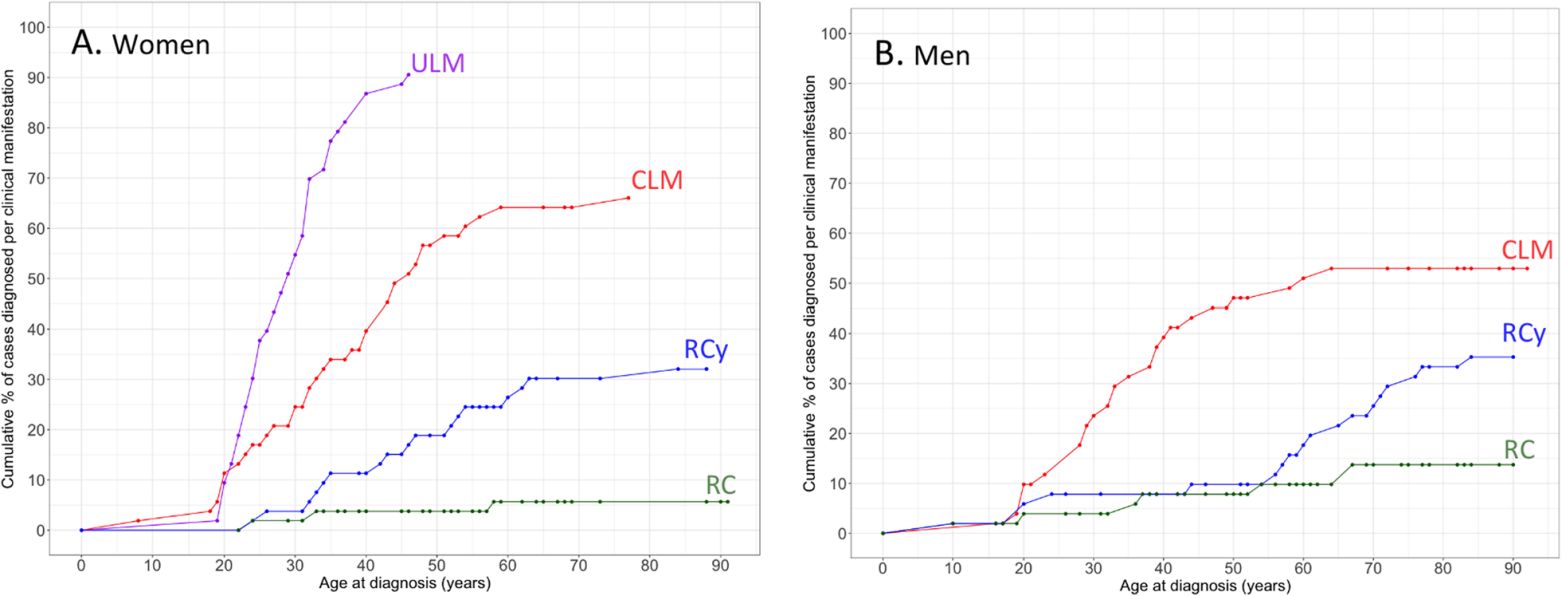
Cumulative incidence based on age at diagnosis of different clinical manifestations in *FH* c.1118A > G carriers according to sex: women (**A**) and men (**B**). Uterine leiomyomas are indicated in purple, cutaneous leiomyomas in red, renal cysts in blue, and renal cell cancer in green

Ninety-nine patients had undergone a dermatological examination and 64 presented CLM (64.6%; 36 women, 28 men). The median age of diagnosis was 35.8 (SD, 13.6) years (Fig. 1). Compared with patients carrying LoF variants, the frequency of CLM was higher in the present cohort (Additional file 3: Table S2). We did not detect differences according to sex. The majority of the relatives were diagnosed with CLM by dermatological examination after positive result on genetic testing. No cases of cutaneous leiomyosarcoma were detected.

Fifty out of the 51 women (98%) who had a gynecological exam had ULM at a median age of 28.7 (SD, 6.8) years (Fig. 1). This frequency was higher than that detected in women with LoF variants (Additional file 3: Table S2). One case was operated at 21 years because of a growing myoma and received a pathological diagnosis of leiomyosarcoma. She had complementary treatment with pelvic radiotherapy. A new pathological review has diagnosed the lesion as an atypical leiomyoma.

RCys were detected in 36 out of the 85 (42.4%) patients who had undergone imaging examinations, 18 (50%) women and 18 (50%) men, at a median age of 50.9 (SD, 19.5) years. These frequencies were higher than those observed in individuals with LoF variants (Additional file 3: Table S2). In a patient diagnosed at 21 years of age with a large renal cyst, the pathological examination showed atypia in the cyst wall.

We confirmed the clinical information in 96 patients, 10 of whom presented with RCC (10.4%; seven men and three women). A woman had two synchronous bilateral RCCs. The median age at diagnosis was 40.6 (SD, 20) years, highlighting three cases with onset at a very young age: a boy at 10 years, a young man at 20 years, and the woman with bilateral RCC at 24 years of age. In addition to papillary type 2 pattern, which was the most frequent histological pattern, other patterns included clear cell renal carcinoma, papillary carcinoma, collecting duct carcinoma, and unclassified carcinoma. There were no differences compared with the patients carrying LoF variant.

## Discussion

HLRCC is a very rare syndrome included in Orphanet’s catalogue of rare diseases [18], with a very low prevalence (1/200,000) [12]. However, it may be underestimated because of misdiagnosis [19, 20]. The main manifestations of HLRCC are CLM and ULM. Skin lesions sometimes are sparse and individuals may think they are stings or acne scars. Moreover, uterine fibroids are relatively frequent in middle-aged women. Therefore, health professionals usually do not suspect that individuals may suffer from HLRCC.

A total of 289 pathogenic and 117 likely pathogenic *FH* variants causing HLRCC or FMRD have been reported in the ClinVar database [ [21], accessed on 04/23/2022)]. The cumulative population frequency of these pathogenic and likely pathogenic *FH* variants is 168.54e−6 (64.43e−6 for LoF and 104.11e−6 for missense variants) [22, 23].

We previously reported a large series of 197 individuals with a genetic diagnosis of HLRCC [13]. We found a recurrent pathogenic variant, *FH* c.1118A > G p.(Asn373Ser), and showed that it has a founder effect in the Alicante province, with a prevalence of HLRCC of 6.26/100,000 inhabitants, which is the highest reported to date.

In the present work, we reported a haplotype study of this recurrent pathogenic variant identified in 104 individuals belonging to 31 apparently unrelated families, which revealed a common haplotype shared among them with an approximate size of 0.61–0.82 Mb. Considering the recombination rate around the *FH* locus per generation, the estimated number of generations elapsed since the origin of the founder mutation is between 12 and 26. This means that we can date the origin of the mutation between the years 1370 and 1720, which mostly corresponds to the historical period of the Modern Age in Spain.

We also described the clinical phenotype of 104 patients from these 31 families. In a bibliographic review, we only found one article describing an *FH* founder mutation that includes the haplotype study of different HLRCC families. Chuang et al. reported an *FH* splicing founder mutation (c.905-1G > A) that was identified in four families of Iranian origin with eight affected individuals [24]. No probands with RCC or RCy were encountered among those families.

We detected an association between missense germline pathogenic variants and the HLRCC clinical phenotype. Individuals carrying missense pathogenic variants exhibited a higher frequency of CLM, ULM, and RCy than did carriers of LoF variants.

The present results obtained for carriers of the *FH* c.1118A > G missense variant confirmed the higher frequencies of CLM, ULM, and RCy in these individuals compared with carriers of LoF variants. However, the frequency of RCC was 10.9%, which is lower than the other published series (12.4–34%) [3, 6–8, 12, 25]. This low rate may be more realistic than those of the published series selected based on the diagnosis of RCC. We described three cases with onset at under 25 years of age. The associated RCC is the most serious manifestation, because of its aggressivity. Despite the low frequency of renal cancer, its early age of onset makes it advisable to use magnetic-resonance-imaging as an early diagnostic procedure, and its cost-effectiveness has been demonstrated [26, 27].

One of the inherent limitations of this type of study is the potential variability in the follow-up of individuals carrying the mutation because of the lack of adherence to the recommendations. This may affect the accuracy of the estimation of the associated risks, especially in less severe clinical manifestations.

Studying patients carrying the same pathogenic variant allows a better assessment of the phenotype. This group represents a unique in vivo model in which the metabolic basis of tumor development, as well as the effect of external risk factors for renal cancer can be studied.

The characterization of a founder mutation using a high number of carriers is the best scenario for the definition of a clinical phenotype specifically associated with that alteration, and for the more precise establishment of the risks associated with each of the related clinical manifestations. In addition, the high prevalence of this founder mutation in our population allows a more efficient genetic diagnosis in suspected cases of HLRCC, as this mutation is screened at the beginning of the diagnostic process.

## Conclusions

In the Spanish province of Alicante there is a high prevalence of HLRCC because of the founder mutation *FH* c.1118A > G; p.(Asn373Ser). A haplotype analysis confirmed that families shared a common haplotype, indicating that the recurrent *FH* c.1118A > G variant was inherited from a founder ancestor. We estimated that the variant appeared between the years 1370 and 1720. The patients carrying these missense mutations had a higher frequency of CLM, ULM, and RCy compared with the frequencies described in HLRCC. However, there is no statistically significant differences in the frequency of RCC in individuals with the founder variant versus individuals with LoF variants. In individuals with suspected HLRCC from the province of Alicante, genetic testing by direct analysis of the founder *FH* c.1118A > G; p.(Asn373Ser) mutation may be a faster and more efficient diagnostic tool compared with complete gene sequencing.

### Supplementary Information

**Additional file 1: Fig. S1** Genetic distribution of the 38 polymorphic markers covering 14 Mb around the *FH* c.1118A > G locus. Genetic markers, both SNPs and STRs, are indicated in black, whereas their genetic position is indicated in grey according to GRCh38/hg38. From top to the bottom, each genetic region marked in red is zoomed below.

**Additional file 2: Table S1.** The most recent common ancestor age estimation of families carrying the *FH* c.1118A > G founder mutation (highlighted), as assessed based on single marker method calculations.

**Additional file 3: Table S2.** Phenotype–genotype correlations. Abbreviations: LoF, loss of function; OR, odds ratio; *, χ^2^ test.

## Data Availability

Data sharing not applicable to this article as no datasets were generated or analysed during the current study.

## Abbreviations

cM: CentiMorgan
CI: Confidence interval
CLM: Cutaneous leiomyomas
FH: Fumarate hydratase
HLRCC: Hereditary leiomyomatosis and renal cell cancer
IQR: Interquartile range
LoF: Loss-of-function
Mb: Megabase
ORs: Odds ratio
RCC: Renal cell cancer
RCys: Renal cysts
STR: Short tandem repeats
SNP: Single nucleotide polymorphism
SD: Standard deviation
TMRCA: Time of most recent common ancestor
ULM: Uterine leiomyomas
