# Supplementary figures and images for: Genetic and clinical characterization of a novel FH founder mutation in families with hereditary leiomyomatosis and renal cell cancer syndrome
Source: Orphanet J Rare Dis. 2024 Jan 26;19:26. doi: 10.1186/s13023-024-03017-z (PMC10811853; doi:10.1186/s13023-024-03017-z)

Chromosome 1  
(q42,2-q44)

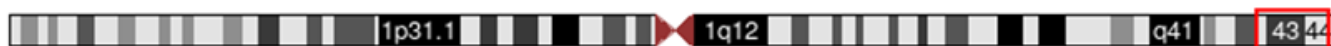

Marker  
Coordinate  
(GRCh38/hg38)

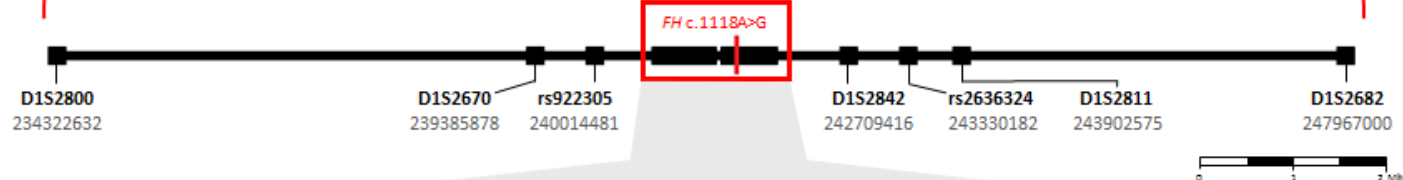

Marker  
Coordinate  
(GRCh38/hg38)

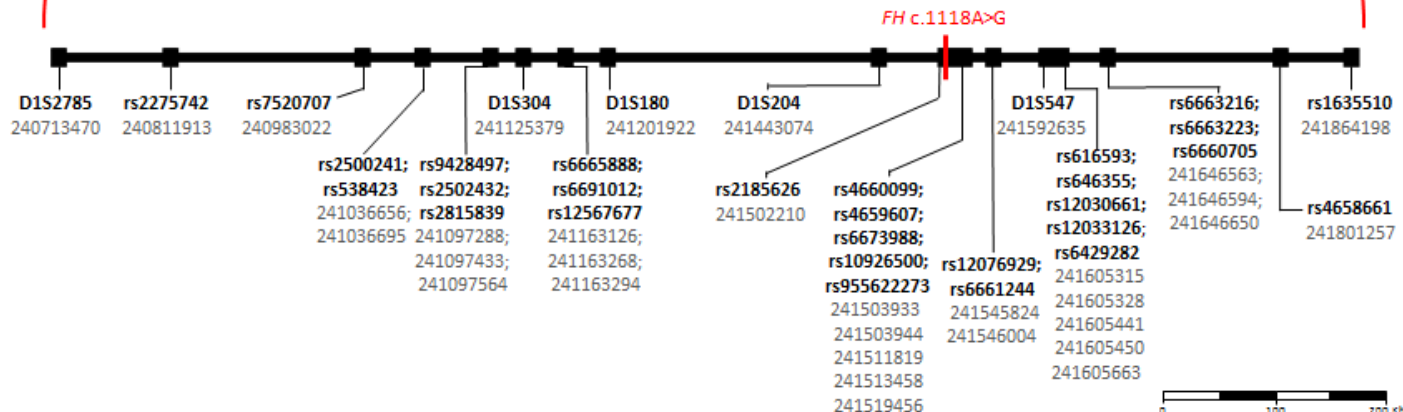

Supplement: Supplementary file 1 — Additional file 1: Fig. S1 Genetic distribution of the 38 polymorphic markers covering 14 Mb around the FH c.1118A > G locus. Genetic markers, both SNPs and STRs, are indicated in black, whereas their genetic position is indicated in grey according to GRCh38/hg38. From top to the bottom, each genetic region marked in red is zoomed below. [file 13023_2024_3017_MOESM1_ESM.pdf]
